# Genetic diversity analysis of angled luffa germplasm resources based on phenotypic traits and resequencing data

**DOI:** 10.3389/fpls.2026.1774008

**Published:** 2026-04-20

**Authors:** Zheng Zeng, Li Zhang, Yuanyuan Guo, Yang Li, Qin Chen, Huanzong Song, Jieling Deng, Xiaoyan Sun, Guijun Su, Xinxin He, Dexian Kang, Zhendong Chen

**Affiliations:** 1 Flower (Facility Agriculture and Leisure Agriculture) Research Institute, Guangxi Academy of Agriculutural Sciences, Nanning, Guangxi, China; 2 Guangxi Fruit and Vegetable Industry Technology Innovation Center, Nanning, Guangxi, China

**Keywords:** angled luffa (*Luffa acutangula* Roxb.), genetic diversity, germplasm resources, phylogenetic evolution, whole-genome resequencing

## Abstract

**Introduction:**

Phenotypic evaluation and whole-genome resequencing (WGR) techniques play critical roles in the identification and utilization of crop germplasm resources. This study integrated 28 phenotypic traits and WGR data to assessphenotypic variation and genetic diversity among 209 angled luffa (Luffa acutangula) accessions.

**Methods:**

A total of 209 angled luffa accessions were evaluated using 28 phenotypic traits and whole-genome resequencing. Phenotypic variation was assessed by coefficients of variation and Shannon-Wiener diversity indices. Principal component analysis (PCA), population structure analysis, phylogenetic tree construction, and hierarchical clustering were performed to analyze phenotypic and genetic diversity.

**Results:**

Extensive phenotypic variation was observed, with coefficients of variation for 20 quantitative traits ranging from 8.41% to 54.09%, and Shannon-Wiener diversity indices (H') from 1.79 to 2.51. For 8 quality-related traits, coefficients of variation ranged from 4.93% to 45.11%, with H' values between 0.13 and 1.97. Principal component analysis revealed 9 principal components, with fruit length and skin color exhibiting the highest loadings, enabling the identification of 12 superior germplasm accessions. Genetic diversity indices indicated limited overall genetic variation (Ne = 1.390, I = 0.528, Ho = 0.125, He = 0.236) with a high inbreeding coefficient (FIS = 0.470), indicating significant inbreeding within this population. Population structure, phylogenetic tree, and hierarchical clustering analyses consistently partitioned the accessions into six subgroups, with each subgroup displaying similar phenotypic and genetic characteristics.

**Discussion:**

Together, these findings provide an integrated phenotypic and genomic landscape of ridged loofah diversity and have laid a solid foundation for the innovative utilization, molecular genetic improvement, and new variety selection of angled luffa germplasm resources in the future.

## 1 Introduction

Angled luffa (*Luffa acutangula* L.) is an annual climbing vine in the Cucurbitaceae family, native to subtropical regions. It was introduced to China in the 6th century and is currently cultivated in various regions of the country (Cai, 2006; Su et al., 2009; Zhang and Wang, 2021). In production, luffa is classified into two cultivated varieties: sponge gourd and angled luffa (Pootakham et al., 2021). Compared to sponge gourd, angled luffa exhibits distinct morphological and postharvest traits, including prominent longitudinal ridges, enhanced strong and transport resilience, and reduced browning. It is predominantly distributed in southern China (Zhu et al., 2024; Lou et al., 2006). The immature fruit of angled luffa is crisp, juicy, and slightly sweet, and is rich in vitamin C, dietary fiber, and essential minerals, making it a nutritionally valuable vegetable (Liu and Guo, 2023; Jiang, 2025). Mature fruits develop extensive vascular fibers and a tough texture, which can be processed into durable, biodegradable products such as bath sponges and kitchen scrubbers, offering high economic and practical utility (Chen, 2023a; Gong et al., 2024; Guo et al., 2025). Its seeds, fruits, vines are used in traditional medicine and contain bioactive compounds, with heat clearing and detoxifying, diuretic and anti-inflammatory properties (Singh et al., 2017; Han et al., 2025; Zhang et al., 2025).

Germplasm resources are considered a strategic foundation of agriculture and an irreplaceable basis for genetic improvement and varietal innovation (Hu et al., 2022; Li et al., 2023; Lv et al., 2025; Xue et al., 2025). China has collected and preserved over 500 luffa germplasm accessions, comprising 462 common luffa (*Luffa cylindrica*) and more than 40 angled luffa (*Luffa acutangula*) varieties (Zhou, 2013; Cao et al., 2025). The breeding research of angled luffa started relatively late, and the evaluation of germplasm quality and genetic research of traits are still limited. Guo et al. (2024) characterized 44 angled luffa accessions using 11 phenotypic parameters. The coefficients of variation ranged from 3.54% to 80.00%, and the genetic diversity index ranged from 1.76 to 2.00, revealing substantial genetic diversity and breeding potential within this germplasm. Chen et al. (2025) comprehensively characterized 14 fruit morphological traits across 30 angled luffa accessions and identified 10 superior lines with exceptional fruit appearance, single fruit weight, and commercial value. Notably, traditional evaluation methods relying on single traits and small sample sizes often fail to comprehensively reflect the overall trait performance of germplasm resources. As the number of collected accessions continues to increase, large-scale conservation, evaluation, and utilization efforts are urgently needed, along with the development of a systematic and objective framework for comprehensive trait assessment.

Phenotype-based assessments of genetic diversity are often strongly influenced by environmental conditions and plant developmental stages (Wang, 2001; Rouphael and Colla, 2005). Zhang et al. (2008) found that morphological traits such as fruit length and circumference exhibited significant seasonal fluctuations; however, phenotypic measurements taken approximately three days after anthesis effectively minimized environmental interference and improved the accuracy of genetic diversity estimation. With advances in molecular biology, researchers have increasingly employed various molecular markers for genetic diversity analysis and population classification of germplasm resources (Wu et al., 2014). Misra et al. (2017) combined ISSR and DAMD molecular markers with morphological data to investigate genetic variation and phylogenetic relationships among five taxonomic groups of cultivated and wild luffa in India. Similarly, Chuenwarin et al. (2020) analyzed 2,834 SNP loci and revealed moderate genetic diversity among 112 ridged luffa accessions from Thailand, classifying them into six subpopulations, providing a scientific basis for parental selection in breeding programs. In recent years, declining sequencing costs and maturing bioinformatics tools have made whole-genome resequencing (WGS) an ideal approach for genetic diversity studies. Unlike conventional molecular markers, WGS does not require prior primer or probe design and enables comprehensive, unbiased detection of all types of genetic variation across the entire genome, thereby fundamentally elucidating the genetic basis of germplasm resources. In the Cucurbitaceae family, Xie et al. (2019) performed WGS on 46 wax gourd accessions and discovered that wild groups retained abundant genetic variation; they classified the germplasm into four clusters and proposed a “two-step domestication” hypothesis for its evolutionary history. Liu et al. (2025) conducted a similar study on 52 Indian squash accessions, using high-quality variant sites to divide them into seven clusters and revealing complex genetic relationships and rich diversity. In contrast, WGS-based genetic research on ridged luffa remains largely unexplored. However, Pootakham et al. (2021) achieved a breakthrough by generating the first chromosome-level reference genome for ridged luffa using PacBio long-read SMRT sequencing integrated with Chicago and Hi-C technologies. Subsequently, Huang et al. (2024) published a high-quality chromosome-scale genome assembly for the highly inbred line SG261, laying a solid foundation for future resequencing and functional annotation studies.

More importantly, WGS is not only a powerful tool for basic research but also offers effective solutions to critical bottlenecks in the angled luffa industry. Currently, the sector faces severe challenges including disease pressures (Downy mildew and Tomato leaf curl New Delhi virus), climate adaptation limitations (notably heat tolerance coupled with cold sensitivity), inconsistent fruit quality (prone to browning, shape deformities, and poor storability and transportability), and inefficient, time-consuming breeding cycles. Traditional breeding approaches struggle to simultaneously improve multiple traits. In contrast, systematic WGS-based genetic diversity assessments of large germplasm collections can precisely identify core accessions carrying key alleles for disease resistance, heat tolerance, and superior quality. Integrating these findings with genomic selection (GS) and marker-assisted selection (MAS) strategies will enable efficient pyramiding of favorable alleles, accelerate their introgression into elite cultivars, establish robust parental lines, and ultimately drive the development of breakthrough varieties—thereby providing strong support for enhancing productivity, quality, and sustainable development in the ridged luffa industry.

Therefore, this study focuses on 209 angled luffa germplasm resources, integrating whole-genome resequencing data with 28 phenotypic traits to analyze genetic diversity, population genetic structure, and perform comprehensive evaluation at both phenotypic and genotypic levels. The findings provide valuable data support for cultivar identification and the selection of optimal hybrid combinations, aiming to accelerate the genetic improvement of angled luffa.

## 2 Materials and methods

### 2.1 Plant materials

A total of 209 angled luffa germplasm accessions were obtained (**Supplementary Table 1**, **Supplementary Figure 1**), 126 accessions from Guangdong province, including 1 from Huidong, 13 from Guangzhou, 7 from Heshan, 7 from Jiangmen, 2 from Shantou, and 95 from unknown regions). 66 accessions from Guangxi province, including 17 from Hezhou, 2 from Nanning, 1 from Beihai, Guangxi, 2 from TianYang, 34 from unknown regions). 3 accessions from Haikou, Hainan province. 2 accessions from Fujian province (1 from Xiamen, 1 from unknown regions). 1 accession from Guiyang, Guizhou province and 2 accessions from Jiangxi province (1 from NanChang, 1 from XiaJiang). 9 accessions from abroad (8 from Thailand, 1 from Malaysia). These materials were collected and developed by the Facility Vegetable Research Team of the Guangxi Academy of Agricultural Sciences.

### 2.2 Experimental sites

The experiment was carried out from 2024 to 2025 at the vegetable production base in Xixiangtang District, Nanning City, Guangxi (28°82′ N; 108°19′ E). The region has a subtropical monsoon climate, with abundant sunshine, less frost and no snow, an annual average rainfall of 1302 mm, an annual average evaporation of 1264 mm, an annual average temperature of 21.6°C, and an average altitude of 67–86 m above sea level. Agroclimatic parameters for the 2024 and 2025 growing seasons are provided in **Supplementary Figure 2**.

### 2.3 Experimental design

The angled luffa germplasm seedlings were raised in 50-cell trays and transplanted upon development of three true leaves. Ridges were constructed to a height of 20 cm and a width of 1.5 m. Plants were spaced 60 cm apart in single rows, resulting in a planting density of approximately 11,100 plants per mu. Irrigation, fertilization, and pest and disease management followed established local conventional practices. For each material, transplant 10 plants to account for poor growth and plant mortality, with one fruit sampled per plant for investigation, ensuring a minimum of 6 fruits per material. In order to consider environmental changes and ensure the robustness of phenotype data, this survey was conducted for two consecutive years, and the final trait values were derived from the two-year average of the collected data.

### 2.4 Trait investigation

Twenty-eight phenotypic traits were investigated the 209 accessions based on Descriptors and Data Standard for Vegetables Sponge (*Luffa* spp.) (Li and Wang, 2007). 28 phenotypic traits include 4 fruit size related traits, namely weight per fruit (WF), fruit length (FL), fruit diameter (FD), fruit flesh thickness (FFT). 11 fruit appearance and texture related traits, namely fruit firmness (FF), fruit shape (FS), size of fruit mottling (SFM), ribbing color (RC), length of fruit end (LFE), rib depth of fruit (RDF), fruit basal color L * (FBC-L*), fruit basal color a * (FBC-A*), fruit basal color b* (FBC-B*), shape of fruit top (SFT), and shape of fruit end (SFE). There are four fruit quality related traits, namely soluble solid matter content (SSMT), vitamin C content (Vc), total protein content (Pro), and total saponin content (Sap). There are two traits related to early maturity, namely the first female flower bearing node (FFN) and maturity (Mat, total growth duration). There are two disease resistance related traits, namely resistance to downy mildew (DMR) and resistance to tomato leaf curl new delhi virus (ToLCNDVR). The five leaf related traits are leaf length (LL), leaf width (LW), leaf color L* (LC-L*), leaf color a* (LC-A*), leaf color b* (LC-B*). The specific measurement methods for traits are detailed in **Table 1** and **Supplementary Figure 3**, and the evaluation criteria for qualitative traits are shown in **Table 2**.

**Table 1:** Quantitative traits and the associated test methods used in the experiment.

Quantitative traits	Abbreviation	Measurement methods
First female flower bearing node	FFN	The node where the first female flower grows on the main vine
Weight per fruit	WF	The quality of a single commodity melon
Fruit length	FL	The length from the stem to the top of the melon
Length of fruit end	LFE	The distance from the bottom of the melon seed cavity to the end of the melon stem
Fruit diameter	FD	The transverse diameter in the middle of the melon
Rib depth of fruit	RDF	Half of the length of the angled transverse diameter minus the length of the transverse diameter
Fruit flesh thickness	FFT	The maximum thickness from the outer edge of the transverse plane to the outer edge of the medullary cavity
Fruit firmness	FF	Using a firmness tester(Top yunnong GY-4) to measure the firmness of fruit peels
Leaf length	LL	The length from the base of the largest leaf in the middle of the main vine to the tip of the leaf
Leaf width	LW	The width at the widest point of the largest leaf in the middle of the main vine
Soluble solid matter content	SSMT	Using a handheld refractometer(PAL-1) to measure soluble solids content in fruit flesh
Vitamin C content	Vc	Reduced ascorbic acid (AsA)/Vitamin content kit(Suzhou grace biotechnology Co., Ltd)
Protein content	Pro	Protein Content (SP) kit (Suzhou grace biotechnology Co., Ltd)
Saponin content	Sap	Total saponin content assay kit (Suzhou grace biotechnology Co., Ltd)
Fruit basal color L*	FBC-L*	Colorimetric analysis using ST-700d spectrophotometer: L* value represents lightness of melon skin chroma
Fruit basal color a*	FBC-A*	Colorimetric analysis using ST-700d spectrophotometer: a* value represents Red-Green of melon skin chroma
Fruit basal color b*	FBC-B*	Colorimetric analysis using ST-700d spectrophotometer: b* value represents Yellow-Blue of melon skin chroma
Leaf color L*	LC-L*	Colorimetric analysis using ST-700d spectrophotometer: L* value represents lightness of leaf chroma
Leaf color a*	LC-A*	Colorimetric analysis using ST-700d spectrophotometer: a* value represents Red-Green of leaf chroma
Leaf color b*	LC-B*	Colorimetric analysis using ST-700d spectrophotometer: b* value represents Yellow-Blue of leaf chroma

L * represents brightness, with higher values indicating brighter and closer to white; the smaller it is, the darker it is, and the closer it is to black. a* represents the red-green chromaticity (positive values represent red, negative values represent green), and the larger the absolute value, the darker the color. b* represents the chromaticity of yellow and blue colors (positive values indicate yellow, negative values indicate blue), and the larger the absolute value, the darker the color.

**Table 2:** Qualitative traits and the associated test methods used in the experiment.

Qualitative trait	Abbreviation	Measurement standard	Measurement method
Maturity	Mat	1.Very early; 2.Early; 3.Intermediate	Count the number of days from sowing period to harvest period
Ribbing color	RC	1.Light green; 2.Green; 3.Dark green	Eye-measurement
Fruit shape	FS	1.Long club-shaped; 2.Short club-shaped; 6.Spindle-shaped; 7.Sickle-shaped	Eye-measurement
Shape of fruit end	SFE	1.Bottle-neck; 2.Inclined shoulder; 3.Bluntly round	Eye-measurement
Shape of fruit top	SFT	1.Gradually acute; 2.Short broadly acute; 3.Broadly round	Eye-measurement
Size of fruit mottling	SFM	0.Absent; 1.Small; 2.Intermediate; 3.Large	Eye-measurement
Resistance to ToLCNDV	ToLCNDVR	1.High resistant; 3.Resistant; 5.Moderate resistant; 7.Susceptive	Field identification and artificial inoculation
Resistance to downy mildew	DMR	1.High resistant; 3.Resistant; 5.Moderate resistant; 7.Susceptive; 9.High Susceptive	Field identification

### 2.7 Data processing

Data were processed using Microsoft Excel 2019 to calculate the mean (μ) and standard deviation (σ) with the AVERAGE and STDEV.S functions. The coefficient of variation (CV) was computed as the ratio of the standard deviation to the mean, expressed as a percentage. Based on μ and σ, all trait data were classified into seven levels, with each interval defined by specific σ ranges: level 1 (x < μ – 2.5σ, including minimum values), level 2 (μ – 2.5σ ≤ x < μ – 1.5σ), level 3 (μ – 1.5σ ≤ x < μ – 0.5σ), level 4 (μ – 0.5σ ≤ x < μ + 0.5σ), level 5 (μ + 0.5σ ≤ x < μ + 1.5σ), level 6 (μ + 1.5σ ≤ x < μ + 2.5σ), and level 7 (x ≥ μ + 2.5σ). For each level, the number of materials was counted and the relative frequency (Pi) was calculated. Trait diversity was evaluated using the Shannon diversity index formula: *H’* = –∑ Pi ln Pi, where ln denotes the natural logarithm (Yuan et al., 2019). Principal component analysis (PCA) was conducted on the standardized dataset using SPSS 24 software (Qi et al., 2024). We extracted principal components based on the criterion that eigenvalues exceed 1 (Eigenvalue > 1) and calculated the variance contribution rate for each component. The weight coefficient (W_i_) was defined as the proportion of the variance contribution rate of the i-th principal component to the cumulative variance contribution rate: Wi = (Variance contribution rate of the i-th principal component)/(Cumulative variance contribution rate). Here, i denotes the i-th principal component. The composite score (F) was obtained by weighting the scores of each sample on all retained principal components according to their respective weight coefficients and summing them: F = W_1_×F_1_ + W_2_×F_2_ +… + W_k_×F_k_. In this equation, F represents the composite score and k is the number of principal components. Germplasm screening was ultimately conducted based on the composite score (F value). We set F > 1.00 as the screening threshold to retain germplasm with overall performance superior to the population mean (Xue et al., 2025). Alternatively, all germplasm were ranked in descending order of F values, and the top N entries were selected as core elite resources according to specific breeding objectives. Correlation analysis, hierarchical clustering analysis and data visualization were performed using Origin Pro 2024 software (Wei et al., 2026). The pearson correlation coefficient was calculated to quantitatively to assess the linear relationship between two variables. Values near 1 indicate a strong positive correlation, values near -1 indicate a strong negative correlation, and values near 0 indicate no substantial linear relationship. All data were Z-score standardized prior to clustering. Hierarchical clustering employed the complete linkage method (longest distance) with Euclidean distance as the distance metric, based on sample similarity in the multi-dimensional feature space (Chen et al., 2023b).

### 2.4 DNA extraction and library construction

In autumn 2024, tender leaves were collected from the tested angled luffa, and then genomic DNA was extracted using the CTAB method (Gill et al., 2025). The samples were sent to Beijing Novogene Co., Ltd. for quality assessment, DNA samples were sent to Beijing Novogene Co., Ltd. (Beijing, China) for quality assessment. DNA degradation and potential contamination with RNA or protein were evaluated by agarose gel electrophoresis. DNA purity (OD260/280 ratio) was measured using a NanoDrop spectrophotometer, and DNA concentration was accurately quantified using Qubit 3.0. Samples with DNA content ≥ 1.5 µg were selected for library construction. Library was constructed using the NEBNext Ultra II DNA Library Prep Kit. DNA samples that passed quality control were randomly interrupted, and library preparation was completed through end repair, A-tailing, adapter ligation, purification, and PCR amplification. Following library validation, libraries were pooled according to effective concentration and target sequencing depth to meet data output requirements. Following library construction, paired-end sequencing (150 bp) was performed on the Illumina HiSeq platform.

### 2.5 Single nucleotide polymorphism detection and annotation

The raw image data generated by high-throughput sequencing were converted into raw sequencing reads through base calling with the resulting data stored in FASTQ format. Stringent quality control and filtering were subsequently performed: adapter/linker sequences were trimmed; reads with N content exceeding 10% were discarded; and low-quality sequences were removed (bases with quality scores Q ≤ 5 constituting >50% of read length). High-quality reads retained after quality control were aligned to the “LAH047” telomere-to-telomere (T2T) reference genome using the BWA-MEM algorithm (parameters: -t 4 -k 32 -M) (Li and Durbin, 2009). After sorting the aligned reads, the rmdup command was used to remove potential PCR duplicates: by retaining only the read pair with the highest mapping quality when multiple pairs shared identical external coordinates. SNP detection across the population samples was then performed using GATK and SAMTOOLS (Aaron, 2010; Fang et al., 2024; Wang et al., 2019). The main filtering parameters are as follows: DP < 3 (minimum depth of coverage for a SNP in a single sample), MISS<0.1 (maximum allowed missing per SNP), and Maf > 0.05 (minimum minor allele frequency threshold). SNP annotation was performed according to reference genome using the package ANNOVAR (Wang, 2010). Based on the genome annotation, SNPs were categorized in exonic regions (overlapping with a coding exon), intronic regions (overlapping with an intron), splicing sites (within 2 bp of a splicing junction), upstream and downstream regions (within a 1 kb region upstream or downstream from the transcription start site), and intergenic regions. SNPs in coding exons were further grouped into synonymous SNPs (did not cause amino acid changes) or nonsynonymous SNPs (caused amino acid changes).

### 2.6 Genetic diversity, population structure and phylogeny analysis

Population genetic diversity analysis was performed using the populations command in the Stacks package. Genetic differentiation (FST) was calculated using VCFtools v0.1.16 (Petr et al., 2011). The diversity indices analyzed included observed heterozygosity (Ho), expected heterozygosity (He), gene diversity index (Nei), effective allele number (Ne), and Shannon index (SHI). To clarify phylogenetic relationships from a genome-wide perspective, an individual-based neighbour-joining (NJ) tree was constructed based on the p-distance using the software TreeBest (see http://treesoft.sourceforge.net/treebest.shtml URLs). The software MEGA6.0 (http://www.megasoftware.net/) was used for visualizing the phylogenetic trees. Additionally, principal component analysis (PCA) was performed to evaluate genetic structure using the software GCTA (Yang et al., 2011) (http://cnsgenomics.com/software/gcta/mlmassoc.html). And the significance level of the eigenvectors was determined using the Tracey-Widom test. The population genetics structure was examined using an expectation maximization algorithm as implemented in the program ADMIXTURE(version 1.23) (Alexander et al., 2009). The number of assumed genetic clusters (K) ranged from 2 to 8 with 10,000 iterations for each run. The optimal K was selected based on the lowest cross-validation (CV) error; when no clear minimum was observed, the elbow point (where the error decrease rate markedly slowed) was used to determine the most appropriate K. Based on the optimal K value, individual ancestry components were estimated to infer population genetic structure.

## 3 Results

### 3.1 Diversity analysis of quantitative traits

Statistical analysis was conducted on 20 quantitative traits of 209 germplasm resources of angled luffa (**Table 3**, **Supplementary Table 2**). The results show that the coefficient of variation (CV) among these traits ranged from 8.41% to 54.09%. Fruit diameter exhibited the lowest CV (8.41%), contrasting with the highest variability in Vc content (54.09%). The remaining traits, ranked in descending order of CV, were: total protein content, leaf color a*, total saponin content, rib depth of fruit, first female flower bearing node, fruit length, fruit firmness, fruit basal color L*, fruit flesh thickness, leaf color b*, fruit basal color a*, weight per fruit, leaf width, length of fruit end, fruit basal color b*, soluble solids content, leaf color L*, and leaf length. Overall, the germplasm resources of angled luffa exhibit relatively rich genetic diversity and phenotypic stability. The Shannon-Wiener index (*H’*) spanned 1.08–2.23 across traits, with total protein content displaying maximum genetic heterogeneity (*H’* = 2.23), while leaf color a* showed constrained variation (*H’* = 1.08), indicating trait-specific selection pressures. The ranking of genetic diversity indices for the remaining traits is as follows: vitamin C content, total saponin content, first female flower node, weight per fruit, fruit diameter, soluble solid matter content, rib depth of fruit, fruit basal color a*, length of fruit end, leaf length, fruit firmness, fruit basal color b*, fruit length, leaf color b*, fruit basal color L*, fruit flesh thickness, leaf color L* and leaf width.

**Table 3:** Analysis of quantitative traits of angled luffa.

Traits	Mean ± SD	Max.	Min.	CV(%)	*H’*
First female flower bearing node	20.28 ± 4.53	38.00	7.10	22.33	2.08
Weight per fruit	326.23 ± 44.11	465.80	223.95	13.52	2.08
Fruit length	35.26 ± 7.06	63.12	23.96	20.01	1.98
Length of fruit end	2.83 ± 0.32	3.79	1.70	11.43	2.05
Fruit diameter	44.98 ± 3.78	55.75	35.83	8.41	2.08
Rib depth of fruit	2.37 ± 0.58	4.17	0.69	24.42	2.07
Fruit flesh thickness	6.37 ± 0.96	10.48	3.61	15.13	1.90
Fruit firmness	4.19 ± 0.70	7.00	2.38	16.74	2.03
Leaf length	24.99 ± 2.11	30.16	18.12	8.46	2.05
Leaf width	25.53 ± 2.99	43.86	17.25	11.71	1.82
Soluble solid matter content	3.73 ± 0.35	4.74	2.90	9.28	2.07
Vc content	12.57 ± 6.80	34.76	1.01	54.09	2.18
Total protein content	7.99 ± 3.82	27.13	1.43	47.88	2.23
Total saponin content	2.89 ± 0.78	6.07	0.68	27.19	2.09
Fruit basal color L*	38.63 ± 6.14	55.82	11.21	15.89	1.92
Fruit basal color a*	-9.74 ± 1.36	-6.72	-15.32	13.99	2.07
Fruit basal color b*	25.93 ± 2.75	35.80	13.93	10.60	2.01
Leaf color L*	37.06 ± 3.24	49.85	24.97	8.74	1.90
Leaf color a*	-9.95 ± 1.56	-6.83	-17.41	15.68	1.08
Leaf color b*	21.27 ± 3.23	36.62	14.10	15.19	1.98

SD, Standard Deviation; Max., Maximum values; Min., minimum value; CV, coefficient of variation; *H’*, Shannon-Wiener Index.

### 3.2 Diversity analysis of qualitative traits

Statistical analysis of 8 qualitative traits was performed on 209 germplasm resources (**Table 4**). The results indicated that the coefficient of variation (CV) ranged from 4.93% to 45.11%. The shape of fruit end exhibited the smallest CV (4.93%), whereas resistance to downy mildew exhibited the largest CV (45.11%). The Shannon-wiener index (*H’*) for qualitative traits ranged from 0.08 to 1.97, with shape of fruit end displaying the lowest diversity (*H’* = 0.08) and resistance to downy mildew recording the highest diversity (*H’* = 1.97). In the tested angledluffa germplasm, fruit end shape, fruit top shape and fruit shape exhibited high frequency distributions, representing 99%, 72%, and 69% of accessions, respectively. In terms of ribbing color, dark green and light green were predominant phenotypes, with the same distribution frequency of 39% for both. For resistance to viral disease (ToLCNDV), 73% of accessions were susceptible. Observed resistance to downy mildew, 78% of the materials were classified as moderately resistant or above.

**Table 4:** Analysis of quantitative traits of angled luffa.

Traits	CV(%)	*H’*	Distribution frequency(%)									
			0	1	2	3	4	5	6	7	8	9
Maturity	36.33	1.26	–	42.93	51.22	5.85	–	–	–	–	–	–
Ribbing color	44.48	1.53	–	39.64	21.36	39.00	–	–	–	–	–	–
Fruit shape	31.79	1.08	–	27.20	68.60	2.40	1.80	–	–	–	–	–
Shape of fruit end	4.93	0.08	–	0.97	99.03	–	–	–	–	–	–	–
Shape of fruit top	24.47	1.08	–	9.70	72.00	18.30	–	–	–	–	–	–
Size of fruit mottling	42.49	1.84	8.91	34.02	42.85	23.13	–	–	–	–	–	–
Resistance to ToLCNDV	40.41	1.53	–	1.90	–	25.12	–	54.98	–	18.01	–	–
Resistance to downy mildew	45.11	1.97	–	5.53	–	41.21	–	31.66	–	13.07	–	8.54

CV, Coefficient of variation; *H’*, Shannon-Wiener Index.

### 3.3 Analysis of variance of quantitative traits

Based on the ANOVA of multi-accession germplasm evaluated over two years, the major agronomic and quality traits of angled luffa were significantly affected by both year (environmental) effects and varietal (genetic) effects, and significant genotype × environment interactions (Year × Variety) were prevalent (**Table 5**). For most traits, the year effect constituted the largest source of variation, with particularly strong effects on fruit length, weight per fruit, first female flower bearing node, leaf length, rib depth of fruit, fruit basal color L* and b*, indicating that these traits are highly sensitive to interannual variation in climate and cultivation conditions. Therefore, varietal evaluation and selection for these traits should be based on multi-environment replicated trials. Varietal effects were also significant or highly significant for multiple key traits, including fruit length, weight per fruit, length of fruit end, fruit diameter, vitamin C content and so on, demonstrating the presence of exploitable genetic variation within the population and providing a solid basis for improving yield, quality, and nutritional value. Notably, Year × Variety interaction effects were significant for most traits, including fruit length, weight per fruit, length of fruit end, fruit diameter, and first female flower bearing node, suggesting differential responses of genotypes to annual environmental conditions. In contrast, the genetic improvement potential of certain traits appeared limited. Although flesh thickness (FFT) showed a highly significant year effect (F = 173.43**), neither the varietal effect nor the interaction effect was significant (F = 1.10 and 1.09, respectively). Likewise, fruit basal color a* showed no significant effects for any source of variation, indicating that under the present germplasm set and test environments, selectable genetic variation for these two indices was insufficient, or that genotypic differences were masked by environmental variation, thereby constraining their potential for genetic improvement.

**Table 5:** Variance analysis of quantitative traits in 209 angled luffa accessions from different years.

Traits source of variation		Year	Variety	Year×Variety	Error
FL	MS	11797.61	576.69	72.07	23.75
	F-value	496.78**	24.28**	3.04**	
WF	MS	1893444.82	22577.03	16615.09	5314.44
	F-value	356.28**	4.25**	3.13**	
LFE	MS	11.96	1.53	1.09	0.39
	F-value	30.65**	3.92**	2.79**	
FD	MS	94.91	178.40	86.66	26.06
	F-value	3.64	6.85**	3.33**	
FFN	MS	31924.07	163.54	67.12	14.15
	F-value	2256.93**	11.56**	4.75**	
RDF	MS	652.36	3.29	2.73	1.26
	F-value	519.21*	2.62*	2.17*	
FFT	MS	12175.54	76.92	76.38	70.21
	F-value	173.43**	1.10	1.09	
FF	MS	7.90	6.01	2.34	0.58
	F-value	13.64**	10.38**	4.04**	
LL	MS	2859.65	61.71	46.37	8.88
	F-value	321.83**	6.94**	5.22**	
LW	MS	2761.98	118.25	104.48	59.78
	F-value	46.20**	1.98**	1.75**	
SSMT	MS	6.36	2.13	1.29	0.22
	F-value	28.79**	9.64**	5.85**	
Vc	MS	447.88	223.27	182.60	38.35
	F-value	11.68**	5.82**	4.76**	
Pro	MS	42.60	2.33	1.43	0.23
	F-value	185.87**	10.17**	6.22**	
Sap	MS	21.30	7.38	6.08	0.79
	F-value	26.91**	9.32**	7.67**	
FBC-L*	MS	38951.57	370.33	101.66	14.81
	F-value	2629.45**	25.00**	6.86**	
FBC-A*	MS	155.98	122.01	110.73	70.08
	F-value	2.23	1.74	1.58	
FBC-B*	MS	14889.24	73.71	76.78	14.81
	F-value	1005.31**	4.98**	5.18**	
LC-L*	MS	3614.94	2645.18	2723.82	2719.73
	F-value	13.39**	0.97	1.00	
LC-A*	MS	176.02	25.50	35.13	1.58
	F-value	111.17**	16.10**	22.19**	
LC-B*	MS	298.19	128.16	122.27	30.31
	F-value	9.84**	4.23**	4.03**	

* indicates significant difference at the 0.05 level, ** indicates significant difference at the 0.01 level.

### 3.4 Correlation analysis among traits of angled luffa

**Figure 1:**
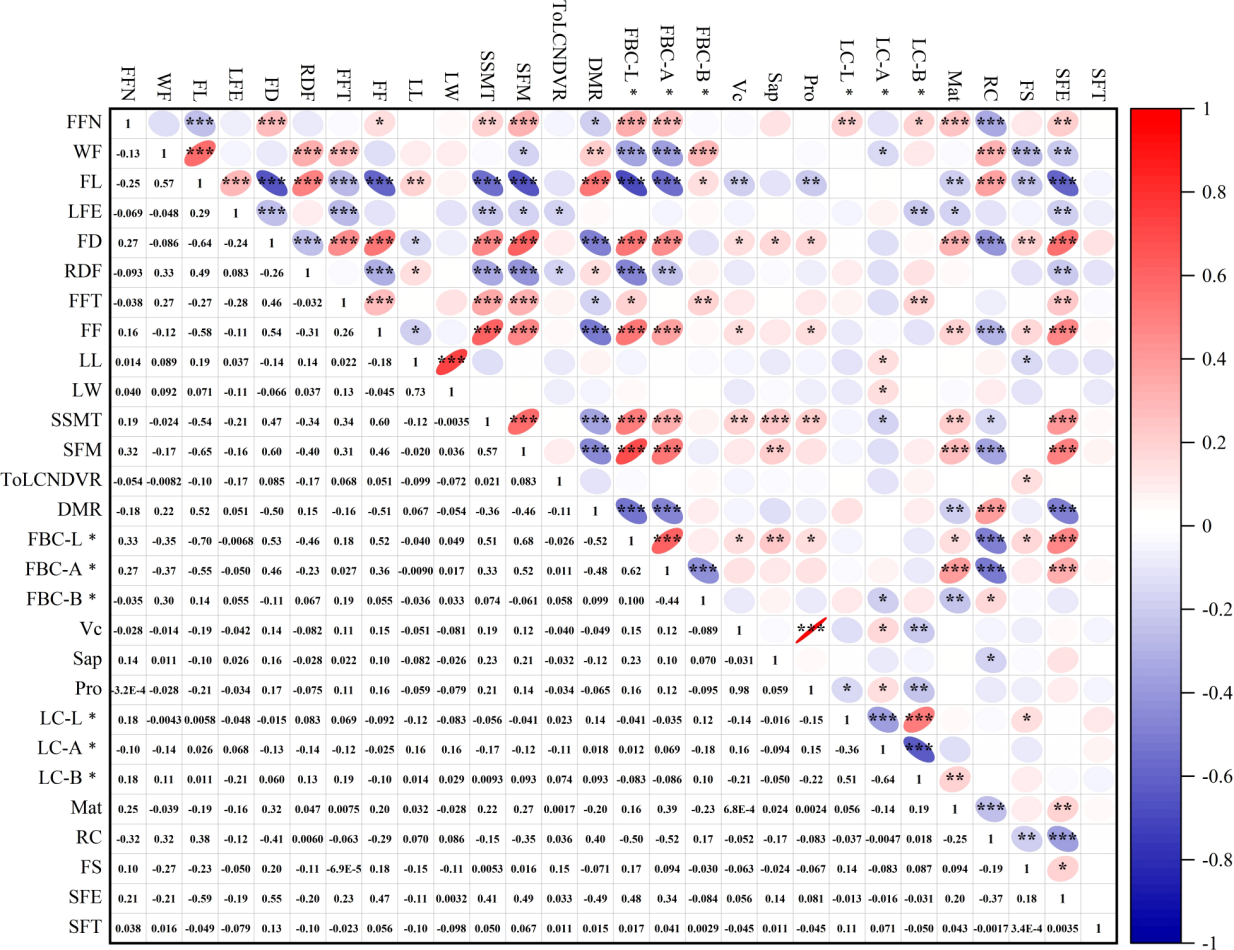
Correlation analysis between phenotypic traits of angled luffa resources. Correlation analysis of phenotypic traits in angled luffa. FFN, first female flower bearing node; WF, weight per fruit; FL, fruit length; LEF, length of fruit end; FD, fruit diameter; RDF, rib depth of fruit; FFT, fruit flesh thickness; FF, fruit firmness; LL, leaf length; LW, leaf width; SSMT, soluble solid matter content; SFM, size of fruit mottling; ToLCNDVR, resistance to ToLCNDV; DMR, resistance to downy mildew; FBC-L*, Fruit basal color L*; FBC-A*, Fruit basal color a*; FBC-B*, Fruit basal color b*; Vc, vitamin C content; Sap, total saponin content; Pro, total protein content; LC-L*, Leaf color L*; LC-A*, Leaf color a*; LC-B*, Leaf color b*; Mat, maturity; RC, ribbing color; FS, fruit shape; SFE, shape of fruit end; SFT, Shape of fruit top. Significance levels are denoted by asterisks: * indicates p < 0.05 (significant), ** indicates p < 0.01 (highly significant), and *** indicates p < 0.001 (very highly significant); ns indicates no significant difference (p ≥ 0.05).

Correlation analysis was conducted on 20 quantitative and 8 qualitative traits across 209 angled luffa accessions (**Figure 1**). In terms of fruit size and yield, single fruit weight showed a significant positive correlation with fruit length (r = 0.57) and fruit flesh thickness (r = 0.27). In contrast, fruit length was significant negative correlated with fruit flesh thickness (r = -0.27), revealing a complex interplay between growth characteristics. In terms of fruit quality, protein content and vitamin C content exhibited a highly significant positive correlation (r = 0.98). Soluble solids content was highly significant positively correlated with saponin content(r = 0.23), and significantly positively associated with fruit diameter (r = 0.47), fruit flesh thickness (r = 0.34), and firmness (r = 0.60). Conversely, soluble solids content was significantly negatively correlated with fruit length (r = -0.54) and edge depth (r = -0.34). In terms of leaves, leaf length and leaf width were significantly positively correlated (r = -0.73), Whereas no significant correlations between leaf dimensions, color and other measured traits. Additionally, the first female flower node was significantly positively correlated with maturity (r = 0.25), fruit diameter (r = 0.27), fruit basal color L*, fruit basal color a*, and fruit mottling size (r = 0.32), but significantly negatively correlated with fruit length (r = -0.25) and ribbing color (r = -0.32). Regarding resistance, no significant correlation was found between antiviral diseases (ToLCNDV) and other measured traits. Resistance to downy mildew was significantly positively correlated with fruit length, and significantly negatively correlated with fruit diameter and firmness. This results indicate that most phenotypic traits in angled luffa germplasm are interactived, often exhibiting mutually reinforcing or compensatory relationships.

### 3.5 Cluster analysis of phenotypic traits of angled luffa

**Figure 2:**
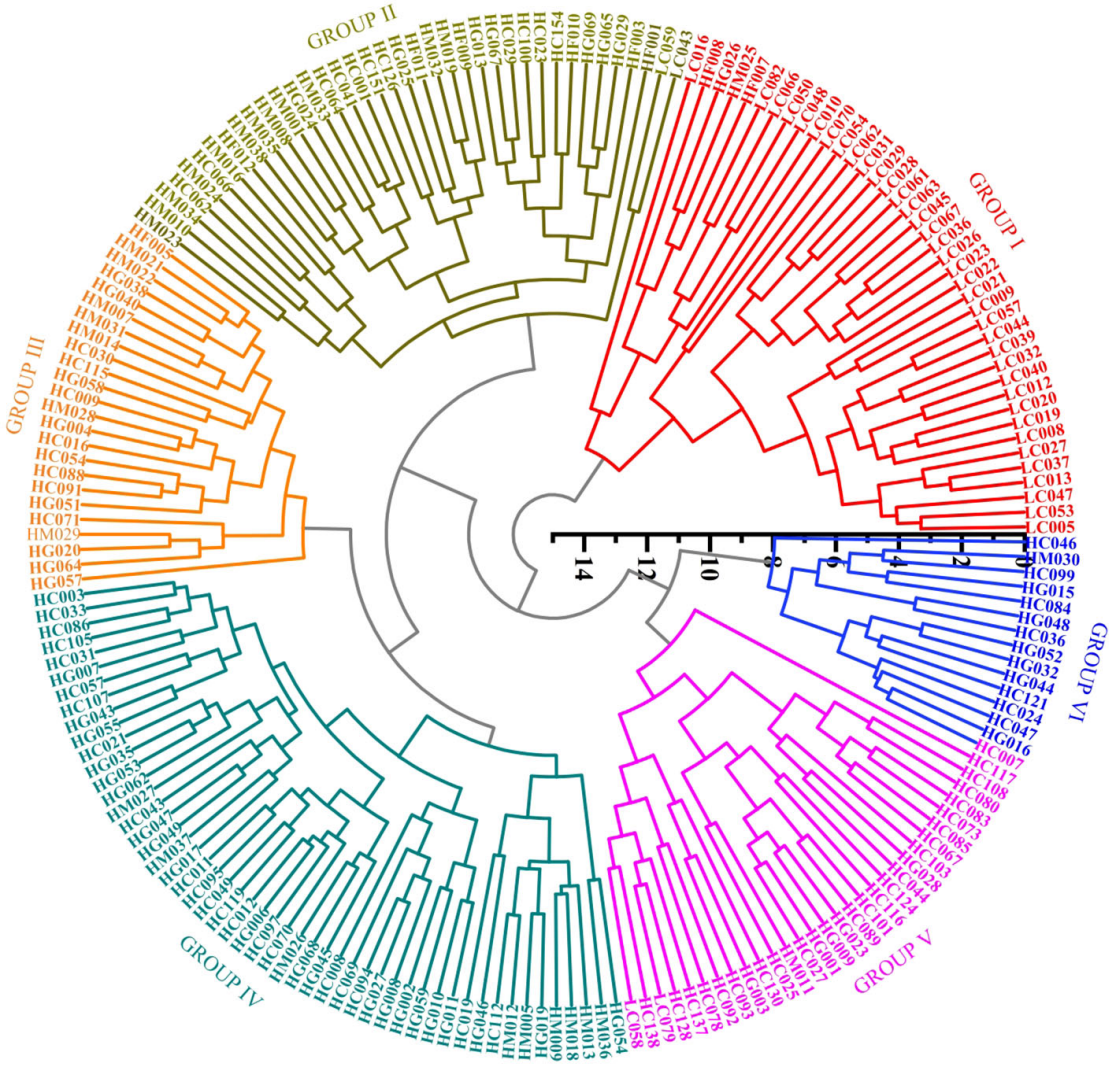
Cluster analysis of phenotypic traits based on angled luffa germplasm resources.

**Figure 3:**
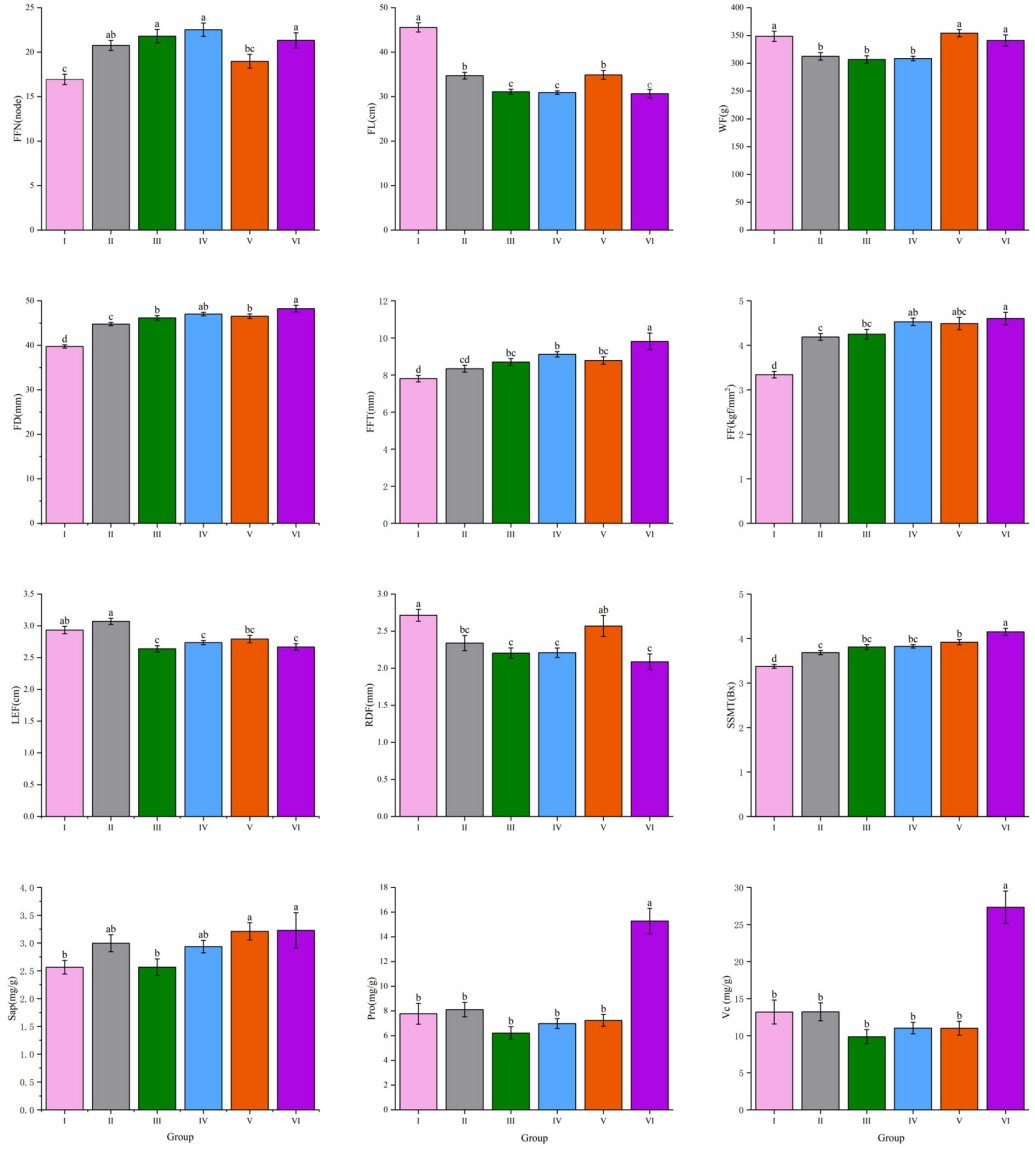
Comparison of important quantitative traits of 6 groups of angled luffa. Different letters represent significant differences between different groups (P<0.01).

A systematic cluster analysis was conducted on 20 phenotypic quantitative traits of angled luffa germplasm resources. The analysis utilized the standardized Ward’s method and calculated Euclidean distances using the complete linkage method to construct a dendrogram. The angled luffa accessions were divided into 6 different groups (**Figure 2**), and the important quantitative traits of each group were compared. The specific results are shown in **Figure 3**. Group I primarily consists of dark green-skinned, long-fruited types, characterized by a fruit length of 45.6 cm, minimal or absent fruit mottling, a low first female flower node at the 16th node, a transverse diameter of 39.0 mm, a thin fruit flesh thickness of 5.9 mm, low firmness (3.3 kgf/mm²), deep fruit rib depth (2.7 mm), low soluble solids content (3.3°Bx) and low saponin content (2.5 mg/g). Group II characterized by a long length of fruit end (3.1 cm), medium fruit length (34.7 cm). Group III is distinguished by a short length of fruit end (2.6 cm), short fruit length (31 cm), low vitamin C content (9.8 mg/100 g), low saponin content (2.5 mg/g) and low protein content (6.2 mg/g). Group IV mainly comprises late-maturing, short-fruited accessions, with key traits of including a short fruit length of 30.9 cm, low single-fruit weight (308.5 g) and high saponin content (3.2 mg/g). Groups V and VII are predominantly composed of accessions with large fruit mottling, thick flesh. Their common characteristics include high single-fruit weight (354.2 g and 340.9 g), large transverse diameter (46.5 mm and 48.2 mm), thick fruit flesh (6.7 mm and 7.2 mm), high firmness (4.4 and 4.6 kgf/mm^2^), high Soluble solid matter content (3.9°Bx and 4.1° Bx) and high saponin content (3.2 mg/g). Specifically, Group V has a medium fruit length of 34.9 cm, while Group VII exhibits a short fruit length (30.6 cm), shallow rib depth of fruit (2.0 mm), high vitamin C content (25.5 mg/100 g) and high protein content of 15.2 mg/g. Angled luffa accessions collected from different regions are generally present in the six groups, and overall clustering analysis shows that they are not significantly correlated with regional sources.

### 3.6 Principal component analysis of phenotypic traits in angled luffa

**Figure 4:**
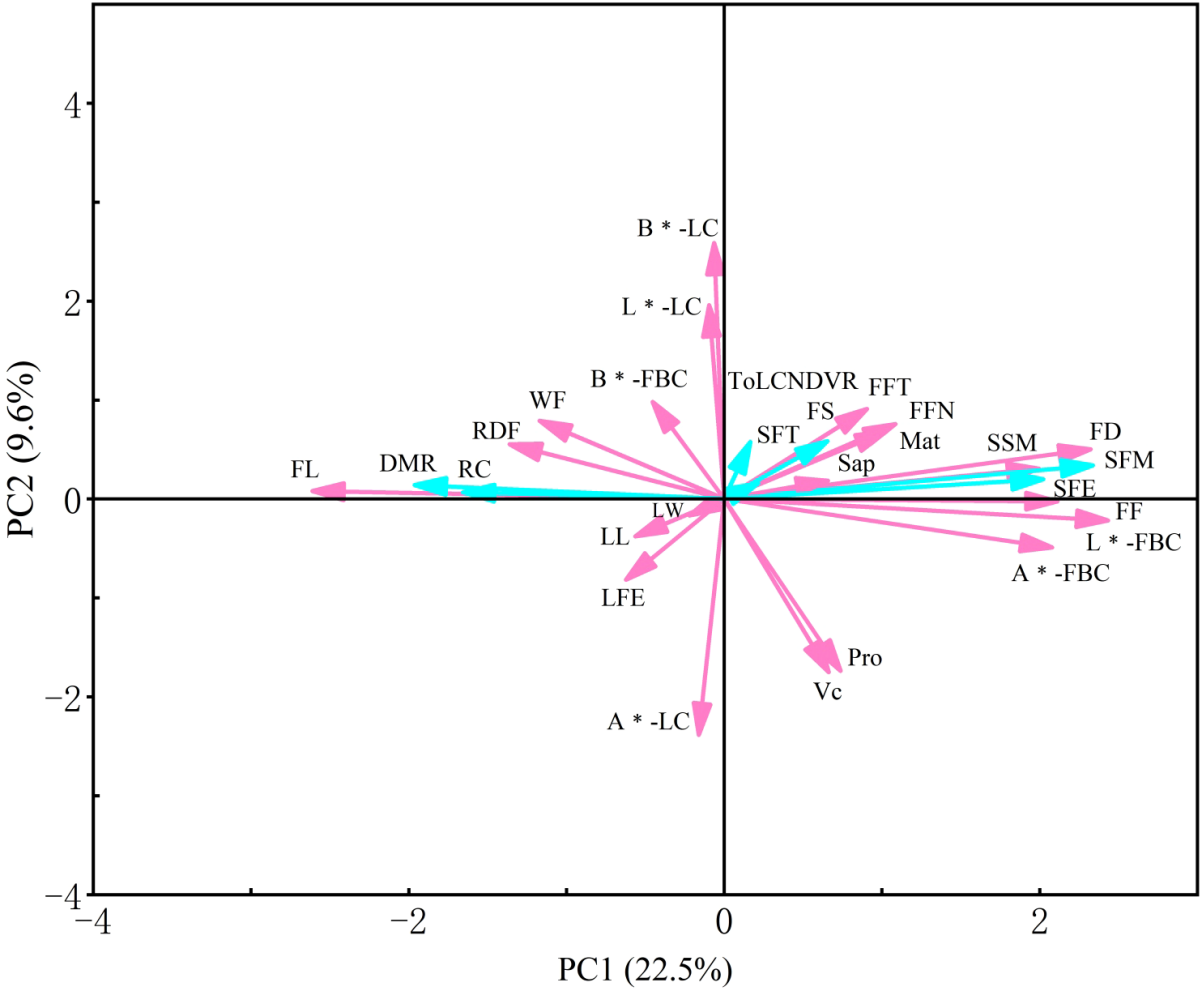
The principal component analysis of the 209 angled luffa accessions. Red represents quantitative traits, while blue represents qualitative traits.

Based on the criterion of eigenvalues greater than 1.0, the first 9 principal components were identified, accounting for 70.048% of the total variance and effectively capturing the majority of information among the 28 phenotypic traits of angled luffa (**Table 6**). Generally, a cumulative variance contribution rate of 70% or higher is considered sufficient to explain the main information of the data. The eigenvalue of PC1 is 6.268, with a variance contribution rate of 22.386%, which was predominantly governed by fruit length, fruit diameter, fruit firmness, fruit basal color, ribbing color, size of fruit mottling, resistance to downy mildew, and soluble solids content. It is a comprehensive reflection of fruit size, taste (mainly manifested as sugar content) and appearance color, reflecting the key goals of breeding improvement and worthy of priority consideration in variety development. PC2 has an eigenvalue of 2.461 and a contribution rate of 8.789%, is mainly related to vitamin C content, protein content and colorimetric parameters L*, a*, b*, indicating that PC2 mainly reflects the nutritional value of the fruit and leaf color related information. The eigenvalue of PC3 is 2.129, with a contribution rate of 7.603%, and it is predominantly driven by single fruit weight and fruit flesh thickness. As a yield related trait, it deserves priority attention in the development of high-yield angled luffa varieties. The eigenvalue of PC4 is 1.925, with a contribution rate of 6.875%, mainly related to leaf length and width, reflecting information related to leaf size. PC6 has an eigenvalue of 1.534 and contribution rate of 5.478%, mainly determined by the first female flower node and fruit rib depth. PC8 has an eigenvalue of 1.275 and a contribution rate of 4.553%, mainly determined by maturity. These two components are key indicators for screening early/late maturing varieties. PC7 has an eigenvalue of 1.347 and a contribution rate of 4.810%, was primarily driven by saponin content and length of fruit end. PC5 and PC9 have eigenvalue values of 1.611 and 1.064, with contribution rate of 5.753% and 3.801%, respectively, and was predominantly governed by fruit shape and shape of fruit end, which was related to the fruit morphology. The relationships between trait indicators and principal components are shown in **Supplementary Figure 4**. Among the quantitative traits (**Figure 4**), fruit length, fruit basal color and fruit diameter contributed the most significantly to PC1 and serve as crucial quantitative trait indicators, among which fruit length is negatively correlated with PC1. For qualitative traits, soluble solids content, vitamin C content and protein content showed positive correlations with PC1 and made significant contributions, serving as essential qualitative trait indicators. Additionally, there is a significant correlation between vitamin C content and protein content. Downy mildew resistance also contributes significantly to PC1, but is negatively correlated with it.

**Table 6:** Principle component analysis of phenotypic traits of angled luffa accessions.

Traits	Principal component									Commonality
	1	2	3	4	5	6	7	8	9	
First female flower bearing node	0.384	0.179	-0.223	-0.03	-0.136	0.421	0.073	0.297	-0.064	0.524
Weight per fruit	-0.338	0.166	0.565	0.156	0.159	0.356	-0.171	-0.127	-0.139	0.702
Fruit length	-0.878	0.009	0.034	0.076	-0.043	0.234	-0.126	-0.056	-0.041	0.855
Length of fruit end	-0.304	-0.186	-0.293	-0.092	-0.229	0.162	-0.422	0.25	0.213	0.585
Fruit diameter	0.791	0.042	0.128	0.013	0.189	0.113	0.023	-0.101	0.08	0.71
Rib depth of fruit	-0.385	0.023	0.033	0.217	-0.014	0.54	-0.125	-0.414	0.327	0.783
Fruit flesh thickness	0.382	0.145	0.519	0.239	0.244	0.073	0.058	-0.234	0.18	0.649
Fruit firmness	0.709	0.003	0.161	-0.009	0.117	-0.107	-0.172	-0.061	0.071	0.593
Leaf length	-0.118	0.021	-0.03	0.796	-0.194	0.058	0.236	0.258	0.11	0.824
Leaf width	-0.02	0.049	0.049	0.811	0.024	-0.113	0.241	0.284	0.116	0.829
Soluble solid matter content	0.682	0.101	0.331	0.013	-0.037	-0.024	-0.12	0.067	-0.28	0.684
Fruit basal color L*	0.832	0.093	-0.03	0.038	-0.088	-0.027	-0.125	0.307	0.067	0.826
Fruit basal color a*	0.616	-0.257	-0.4	0.2	-0.128	0.104	0.102	-0.253	-0.153	0.770
Fruit basal color b*	-0.074	0.482	0.496	-0.055	0.147	0.092	-0.256	0.215	0.283	0.708
Vc content	0.239	-0.582	0.519	-0.214	-0.419	0.12	0.259	0.028	0.122	0.984
Saponin content	0.221	0.027	0.058	-0.026	-0.034	0.253	-0.46	0.201	-0.315	0.471
Protein content	0.267	-0.574	0.518	-0.217	-0.42	0.147	0.211	0.055	0.108	0.974
Leaf color L*	0.035	0.527	-0.06	-0.387	-0.073	0.271	0.363	0.215	0.031	0.69
Leaf color a*	-0.101	-0.614	-0.068	0.017	0.593	0.213	0.145	0.213	0.034	0.856
Leaf color b*	0.104	0.714	-0.005	-0.144	-0.074	0.254	0.352	0.031	-0.033	0.737
Size of fruit mottling	0.783	0.05	0.031	0.12	0.018	0.064	-0.026	0.13	-0.191	0.689
Resistance to ToLCNDV	0.109	0.153	0.05	-0.105	0.233	-0.329	0.197	-0.31	0.042	0.348
Resistance to downy mildew	-0.635	0.049	0.116	-0.172	-0.043	0.057	0.277	0.161	-0.159	0.582
Maturity	0.379	0.065	-0.247	0.108	-0.11	0.38	0.205	-0.417	-0.236	0.648
Fruit shape	0.202	0.137	-0.233	-0.297	0.094	-0.114	0.147	0.062	0.492	0.491
Ribbing color	-0.517	0.109	0.396	0.018	0.039	-0.355	0.152	0.08	-0.334	0.704
Shape of fruit end	0.003	-0.357	-0.047	-0.156	0.71	0.319	0.197	0.222	-0.105	0.86
Shape of fruit top	0.698	0.026	0.009	0.02	0.139	-0.078	-0.084	-0.007	0.133	0.538
Contribution rate (%)	22.386	8.789	7.603	6.875	5.753	5.478	4.810	4.553	3.801	
Total account (%)	22.386	31.175	38.778	45.652	51.406	56.884	61.694	66.247	70.048	

### 3.7 Comprehensive evaluation of germplasm resources of angled luffa

To screen for excellent germplasm resources of angled luffa, 28 standardized phenotypic traits of angled luffa were substituted into 9 principal components to obtain the principal component F score function: F1 = 0.153×FFN-0.135×WF-0.351×FL-0.121×LFE+0.316×FD -0.154×RDF+0.153×FFT+0.283×FF-0.047×LL -0.008×LW+0.272×SSMT+0.332×FBC L*+0.246×FBC A*0-0.029×FBC B*+0.095×Vc+0.088×Sap+0.106×Pro+0.014×LC L*-0.040×LC A*+0.042×LC B*+0.313×SFM+0.044×ToLCNDVR-0.254×DMR+0.151×Mat+0.081×FS-0.206×RC+0.001×SFE+0.279×SFT. Based on the scores of 9 principal components, the variance explained by each principal component was used as a weight for linear weighted averaging, and finally the comprehensive evaluation formula for each germplasm is obtained: F = 0.320×F1 + 0.125×F2 + 0.109×F3 + 0.098×F4 + 0.082×F5 + 0.078×F6 + 0.069×F7 + 0.065×F8 + 0.054×F9. The comprehensive score (F) range for the germplasm resources of angled luffa is -2.826 to 2.032, with higher comprehensive scores indicating better overall performance. The 209 angled luffa accessions were systematically ranked based on F-values, and the top 12 accessions with the highest scores were selected (F > 1.0) (**Table 7**). Detailed scores of accessions are provided in the **Supplementary Table 3**. Higher-rankings accessions are mostly characterized by short fruit, large transverse diameter, large fruit mottling, high soluble solids content, light rind color, thick flesh and relatively high firmness. Correlation analysis between the comprehensive score F and phenotypic traits (see **Supplementary Table 4**) revealed that most traits were significantly or extremely significantly correlated with F value, with 17 traits reaching the extremely significant level and 6 traits reaching the significant level. The F-value is significantly or extremely significantly correlated with 76.7% of the traits. Therefore, the F-value can be used as an important basis for comprehensive evaluation of the germplasm resources of angled luffa.

**Table 7:** Information on important agronomic traits of excellent angled luffa germplasm.

Name	WF (g)	FL (cm)	FD (mm)	FFT (cm)	FF (kgf/mm²)	SSMT (°Bx)	Vc (mg/100 g)	Pro (mg/g)	SFM
HC036	365.74	24.68	54.81	9.48	5.36	4.65	20.34	12.08	4
HC025	390.42	32.78	47.99	10.48	4.34	4.19	9.77	6.04	3
HC007	314.58	28.05	48.87	8.21	5.14	4.20	30.48	16.66	3
HG048	294.84	28.98	49.35	8.99	5.09	4.74	16.88	10.16	4
HC117	340.98	29.13	48.81	4.26	4.69	3.93	12.97	7.99	3
HC084	367.72	32.03	47.13	7.08	4.82	4.54	22.35	13.54	4
HC057	296.31	27.58	47.07	6.97	4.87	3.90	11.22	7.46	5
HG057	285.37	25.39	51.64	7.58	4.56	3.62	12.97	7.99	3
HM027	313.40	30.19	55.75	8.63	4.34	3.67	12.15	7.84	4
HG007	306.37	27.83	48.20	7.29	5.35	3.85	12.88	7.54	5
HC055	299.97	26.47	42.82	6.52	4.89	3.93	6.12	4.86	4
HG014	388.18	32.56	47.94	7.28	4.50	3.86	11.79	7.57	3

WF, weight per fruit; FL, fruit length; FD, fruit diameter; FFT, fruit flesh thickness; FF, fruit firmness; SSMT, soluble solid matter content; Vc, vitamin C content; SFM, size of fruit mottling.

### 3.8 Genetic diversity analysis of angled luffa

**Figure 5:**
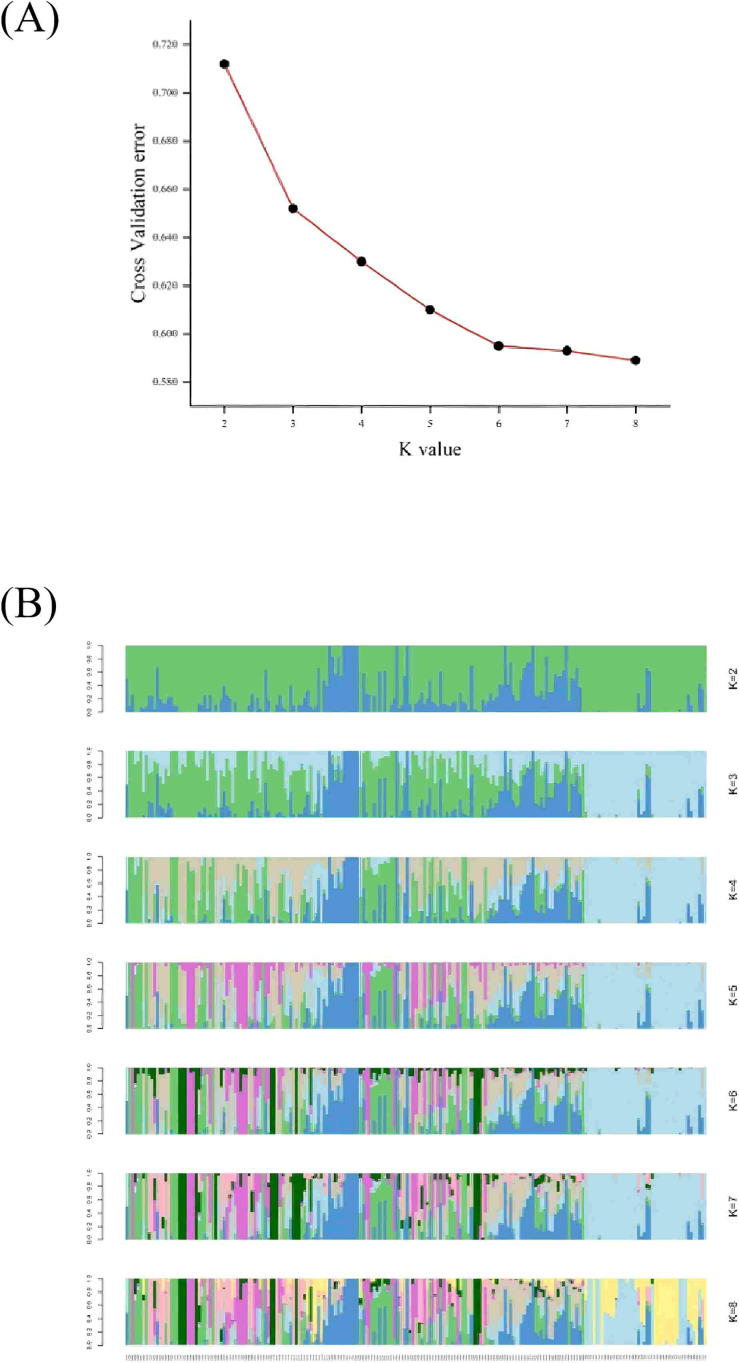
Analysis of population structure of 209 angled luffa varieties. **(A)** CV error distribution chart. **(B)** Population genetic structure graph of 209 angled luffa germplasms based on structure. Numbers in the horizontal axis represent serial number of accessions.

**Figure 6:**
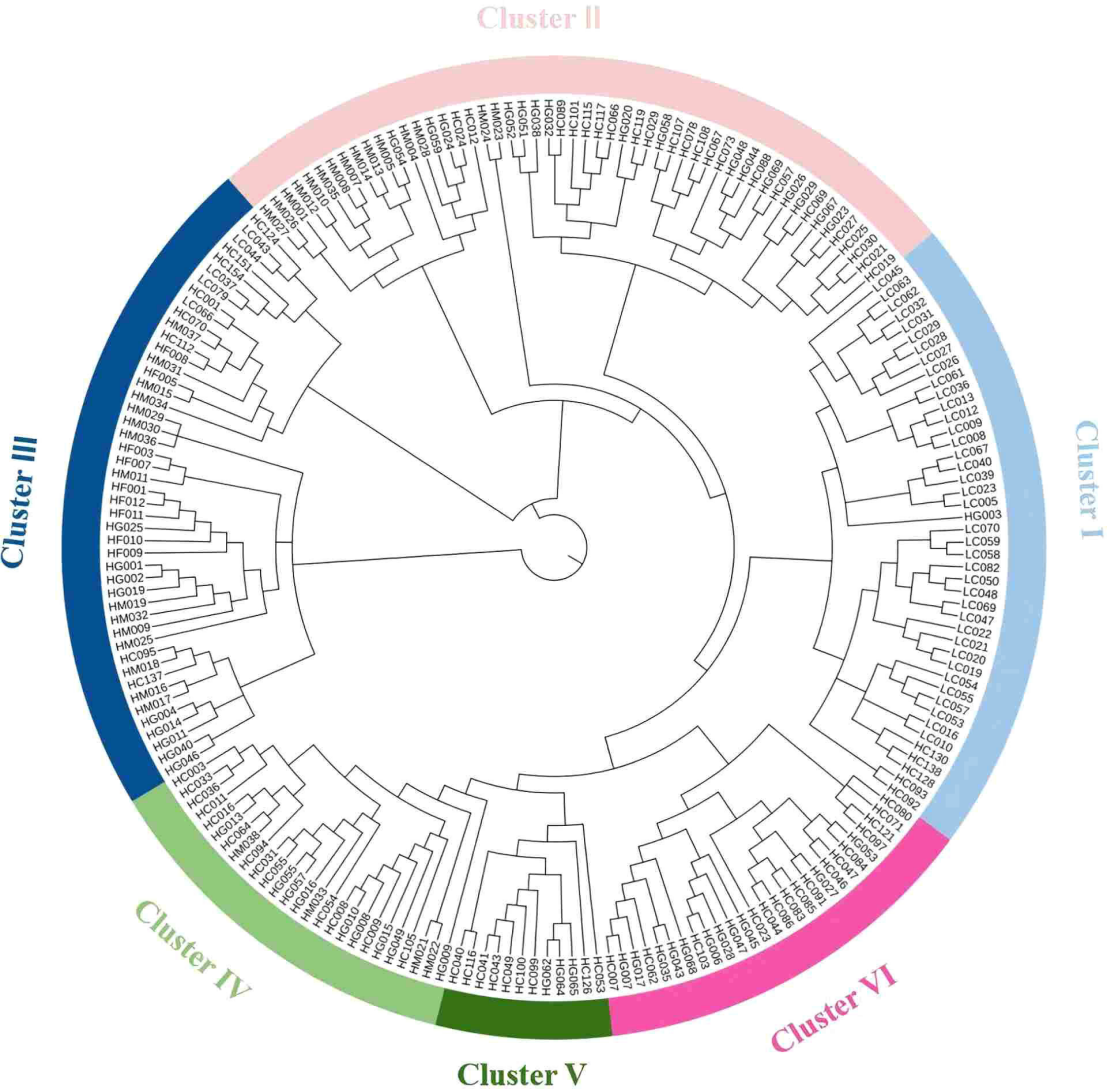
Phylogenetic tree of 209 angled luffa varieties constructed by the neighbor-joining method.

Using the T2T genome of the angled luffa as a reference (unpublished), we re-sequenced 209 accessions (**Supplementary Table 5**) and achieved an average mapping rate of 99.08%, an average depth of 23.23×, an average genome coverage of 94.71%, and a mean GC content of 38.03% (**Supplementary Table 6**). After SNP calling using SAMtools and associated modules, 8,849,942 raw variants were detected; stringent filtering retained 4,264,901 high-quality SNPs, which were functionally annotated (**Supplementary Tables 7**, **8**). Population genetic diversity analysis was conducted using the *populations* module in the Stacks package. As shown in **Table 8**, Cluster 6 exhibited the highest genetic variation, with a Shannon diversity index (I) of 0.71 and nucleotide diversity (Pi) of 0.33. In contrast, Cluster 5 showed relatively lower diversity, with I = 0.39 and Pi = 0.18. The effective number of alleles (Ne) ranged from 1.30 to 1.56, with a mean of 1.40. Observed heterozygosity (Ho) varied between 0.10 and 0.18, while expected heterozygosity (He) ranged from 0.18 to 0.33, with respective averages of 0.13 and 0.24. The mean inbreeding coefficient was 0.46, indicating moderate inbreeding within the population. Therefore, expanding population size and promoting random mating are recommended to mitigate inbreeding depression risks. Pairwise FST values among populations ranged from 0.055 to 0.117 (**Table 9**), suggesting moderate genetic differentiation. Populations 4 and 5 displayed the weakest genetic differentiation and the most frequent gene flow, indicating close genetic relatedness. Conversely, Population 6 showed consistently high Fst values relative to all other populations, reflecting the highest level of genetic differentiation. Consequently, Population 6 should be prioritized for conservation to prevent loss of genetic diversity. To investigate population structure and genetic relationships, we performed genetic structure analysis. The cross-validation error decreased only marginally (ΔCV < 0.01) when K exceeded 6 (**Figure 5A**), indicating that K = 6 is the most biologically plausible clustering solution, consistent with the principle of parsimony (Occam’s Razor) and avoiding overinterpretation of population subdivision. At K = 6, Q1 included 56 accessions from 9 regions, Q2 included 32 accessions from 7 regions, Q3 included 24 accessions from 10 regions, Q4 included 44 accessions from 10 regions, Q5 included 13 accessions from 5 regions, and Q6 included 40 accessions from 6 regions (**Figure 5B**). A phylogenetic tree was constructed using the neighbor-joining method, which classified the 209 angled luffa germplasm accessions into six subgroups (**Figure 6**). Cluster I contained 44 accessions, all from Q1. This group is characterized by long fruit type, early maturity, dark green of fruit basal color, soft flesh texture, and inferior overall quality traits. Cluster II contained 26 accessions, all from Q2. The main features include late maturity and relatively long fruit diameter. Cluster III contained 27 accessions, 23 of which were from Q3. This group exhibits the maximum length of fruit end but the lowest single fruit weight, indicating poor yield performance. Cluster IV contained 53 accessions, 40 of which were from Q4. The distinguishing characteristics are the shallowest ridges and the highest saponin content. Cluster V contained 12 accessions, 11 of which were from Q5. And Cluster VI contained 46 accessions, 36 of which were from Q6. This group represents short fruit type varieties with thick flesh, distinct mottling on the fruit surface, and the highest contents of vitamins, total protein, and soluble solid matter among all six groups (**Supplementary Figure 5**, **Supplementary Table 8**). Both analyses partitioned angled luffa into six subgroups with relatively consistent characteristics. However, due to the close genetic relationships among some accessions, inbreeding and gene flow are common, leading to discrepancies in genomic background and locus assignment. Additionally, substantial environmental fluctuations across years and seasons, combined with differing distance metrics used in phenotypic clustering versus SNP-based phylogenetic inference, collectively contribute to the assignment of identical materials to distinct subclades in phenotypic and molecular trees.

**Table 8:** Variation of genetic parameters in 209 populations of angled luffa.

Populations	He	Ho	I	Ne	Nei	Pi	FIS	FIT
1	0.23	0.12	0.53	1.37	0.11	0.23	0.48	0.53
2	0.22	0.11	0.51	1.36	0.11	0.23	0.50	0.54
3	0.18	0.10	0.40	1.31	0.09	0.18	0.44	0.49
4	0.28	0.15	0.63	1.47	0.14	0.29	0.46	0.49
5	0.18	0.11	0.39	1.30	0.09	0.18	0.39	0.44
6	0.33	0.18	0.71	1.56	0.16	0.33	0.45	0.51
Average	0.24	0.13	0.53	1.40	0.12	0.24	0.46	0.51

He, expected heterozygosity; Ho, observed heterozygosity; I: shannon diversity index, Ne, effective number of alleles; Nei, Nei’s genetic diversity; Pi, nucleotide diversity; PIC, polymorphism information content; Fis, inbreeding coefficient within subpopulations; Fit, total inbreeding coefficient.

**Table 9:** Paired Fst values of 6 subgroups of 209 angled luffa accessions.

Populations	1	2	3	4	5	6
1	0.000					
2	0.088	0.000				
3	0.114	0.073	0.000			
4	0.061	0.064	0.076	0.000		
5	0.081	0.079	0.096	0.055	0.000	
6	0.117	0.102	0.111	0.071	0.104	0.000

## 4 Discussion

Phenotypic variation and diversity are key indicators of genetic diversity in germplasm (Chen et al., 2024; Xue et al., 2025), providing an essential genetic foundation and selection pool for breeding. In this study, 28 phenotypic traits of 209 angled luffa accessions were statistically analyzed. The results showed that CV for quantitative traits ranged from 8.48% (fruit diameter) to 60.07% (vitamin C content), whereas the CV for qualitative traits ranged from 4.93% (fruit end shape) to 45.11% (resistance to downy mildew). These results demonstrate abundant phenotypic variability within the germplasm. Notably, vitamin C content, total protein content, size of fruit mottling and the degree of variation in resistance to downy mildew exhibited relatively high variability, consistent with the findings of Guo et al. (2024) and Qiu et al. (2024). This indicates substantial inter-accession differences and ample selection space; breeders can exploit extreme phenotypes as elite parents to assemble crosses and achieve effective improvement. The Shannon-Weaver diversity index for quantitative traits ranged from 1.08 to 2.23, and for qualitative traits from 0.08 to 1.97. Fruit length, single fruit weight, first female flower node, fruit basal color, fruit firmness, and leaf length showed relatively high diversity indices, agreeing with the results of Guo et al. (2024) and Cao et al. (2025). In contrast, fruit shape, shape of fruit end, and shape of fruit top had low diversity indices, consistent with the research of Qiao et al. (2022) and Chen et al. (2025). Compared with quantitative traits, most qualitative traits in this study (e.g., shape, color) displayed overall lower diversity indices, suggesting that these morphological features experienced stronger selection pressure during domestication, resulting in reduced genetic diversity—aligning with the classical breeding theory proposed by Guo et al. (2024). To broaden the source of variation, future breeding should introduce wild relatives or foreign landraces. Based on these results, traits with rich variation and higher genetic diversity can be subjected directly to genome-wide association studies (GWAS) in the panel of 209 accessions to efficiently detect significant loci underlying nutritional quality, resistance, and agronomic traits. For traits with complex genetic bases or lower diversity, representative accessions with extreme phenotypes can be crossed to build F2 or recombinant inbred line (RIL) populations for QTL mapping and fine dissection of their genetic architecture, thereby enabling marker-assisted selection.

Correlation analysis of phenotypic traits can reveal the degree of association among different characters. When a major trait is targeted for genetic improvement, secondary traits may be improved simultaneously, thereby facilitating the design of efficient breeding schemes and accelerating genetic gain (Zhou et al., 2021; Li et al., 2023). In this study, correlation analyses were conducted for 209 angled luffa accessions and significant associations were detected among most traits. Single fruit weight was positively correlated with fruit length, flesh thickness and rib depth (*P* < 0.01), suggesting that simultaneous increases in these traits could jointly enhance yield. By contrast, fruit length was negatively correlated with flesh thickness, fruit diameter, fruit firmness, mottling size and soluble solid content, which is consistent with the research findings of Su et al. (2009) and Cao et al. (2025), indicating that as fruit length increases, these traits exhibit oppositing trends. In production, the long-fruit type angled luffa commercial fruit is 50–70 cm long and 4–5 cm in diameter, with thin flesh, low firmness, low soluble solids, and large mottling spots. The short-fruit type commercial fruit, measuring 20–30 cm in length and 6–8 cm in diameter, exhibits thick flesh, high sugar content, and small mottling spots, further verifying the practical universality of the negative correlation mentioned above.

Principal component analysis (PCA) can effectively integrate multiple complex traits, extract the main sources of variation, and assist breeders in identifying key traits for research and improvement (Li et al., 2024; Lv et al., 2025; Dong et al., 2025). In this study, 9 principal components (PCs) with eigenvalues greater than 1.0 were extracted from the 28 phenotypic traits, with a cumulative contribution rate of 70.048%, effectively capturing the majority of variation in the germplasm. The nine representative traits identified in this study are basically consistent with the traits prioritized by Qiu et al. (2024), indicating that these traits can serve as universal indicators for germplasm evaluation. To overcome the limitations of single-trait selection, we developed a PCA-based comprehensive evaluation system. Weight coefficients (Wi) were determined as the ratio of each PC’s variance contribution to the cumulative contribution, and a comprehensive score function was established as F = Σ(Wi × Fi) (i = 1, 2, …, 9) to quantify multi-trait performance. Using a threshold of F > 1.00, 12 superior accessions were screened (**Table 6**), displaying clear advantages in yield-related traits and nutritional quality. These elite accessions should be prioritized as core germplasm and provide parent selection for different breeding objectives.

Based on whole-genome SNP data, this study systematically analyzed the genetic diversity and population structure of angled luffa. Heterozygosity is an effective indicator of population genetic diversity and positively correlates with overall genetic variation. In this study, the expected heterozygosity (He) was 0.24, and the observed heterozygosity (Ho) was 0.13, and the inbreeding coefficient (FIS) was 0.46, indicating relatively low genetic diversity and a pronounced inbreeding effect. This likely reflects the narrow geographic origin of the materials (predominantly from South China) and long-term regional selection that increases the probability of consanguineous mating. In addition, market-oriented breeding often targets traits such as fruit shape and color; while enhancing these commercial traits, diversity is inadvertently reduced. Therefore, it is urgent to expand population size, particularly by introducing wild relatives or foreign landraces, performing wide crosses to broaden the gene pool, promoting random mating within conservation populations, and gradually lowering the inbreeding coefficient. Genetic structure analysis revealed six distinct genetic clusters, a finding consistently supported by principal component analysis (**Supplementary Figure 3**) and phylogenetic tree reconstruction. Both cluster analysis and the phylogenetic tree classified the germplasm into six groups, with phenotypic characteristics highly consistent across groupings. The accessions in Group 1 (corresponding to Cluster 1) are predominantly deep green-skinned, melon-shaped, and slender, with no or minimal fruit mottling, early maturity, and pronounced ribbing. Group 2 (corresponding to Cluster 3) exhibits long pedicels and dark fruit margins. Group 3 (corresponding to Cluster 4) demonstrates advantages in leaf size. Group 4 (corresponding to Cluster 2) consists primarily of late-maturing accessions. Group 6 (corresponding to Cluster 6) includes light green-skinned, short melon-type accessions with thick flesh, extensive fruit mottling, and the highest levels of vitamins, total protein, saponins, and soluble sugars—key quality and nutritional components. Thus, Group 6 may serve as preferred parental material for high-quality breeding programs. Group 5 (corresponding to Cluster 5) displays moderate performance across all phenotypic traits. The concordance of results across multiple analytical methods indicates a relatively stable population structure, enhancing the reliability of the findings and providing a clear genetic framework for breeding applications. Based on genetic distance, Group 5 and Group 4 showed the smallest differentiation (FST = 0.055), indicating highly similar genetic backgrounds; one subgroup or representative accessions can be retained for conservation, and crosses between these two subgroups should be avoided. In contrast, Group 6 displayed large differentiation from all other groups and should be prioritized as core germplasm. We recommend using Group 6 as a preferred parent in intergroup crosses with more distant groups such as Group 1 and Group 3 to maximize heterosis via higher inter-subgroup genetic distances and to broaden the genetic base. For different improvement goals, parents with complementary trait advantages can be selected from specific subgroups, while leveraging larger intergroup FST to increase offspring heterozygosity and heterosis.

## 5 Conclusion

This study comprehensively evaluated the phenotypic diversity of 209 angled luffa germplasm resources, encompassing 28 traits related to fruit yield, appearance, and nutritional quality. The results indicate substantial phenotypic variation among the accessions. A total of 12 elite germplasm lines were identified, which fulfill the requirements for germplasm development and genetic breeding programs. Based on whole-genome resequencing data, the genetic diversity within the angled luffa population was found to be limited, highlighting the need to expand the collection of wild accessions and geographically diverse varieties to enhance the genetic base of existing resources. Furthermore, clustering analysis, Admixture analysis, and phylogenetic tree analysis consistently partitioned the population into six distinct subgroups, each exhibiting shared phenotypic characteristics. This concordance across analytical methods confirms the robustness of the population structure inference. Subsequently, genome-wide association study (GWAS) analyses integrating key trait data can be performed to identify candidate genomic regions and causal mutations underlying important agronomic traits, thereby providing a solid theoretical foundation for marker-assisted breeding and gene discovery in angled luffa.

## Data Availability

The raw data for the reference genome used in this study have been deposited in NCBI under accession number PRJNA1450820.
