# Supplementary material for: Genetic diversity analysis of angled luffa germplasm resources based on phenotypic traits and resequencing data
Source: Front Plant Sci. 2026 Apr 20;17:1774008. doi: 10.3389/fpls.2026.1774008 (PMC13136258; doi:10.3389/fpls.2026.1774008)
Supplement: Supplementary file 2 [file Presentation1.pptx]

## Slide 1
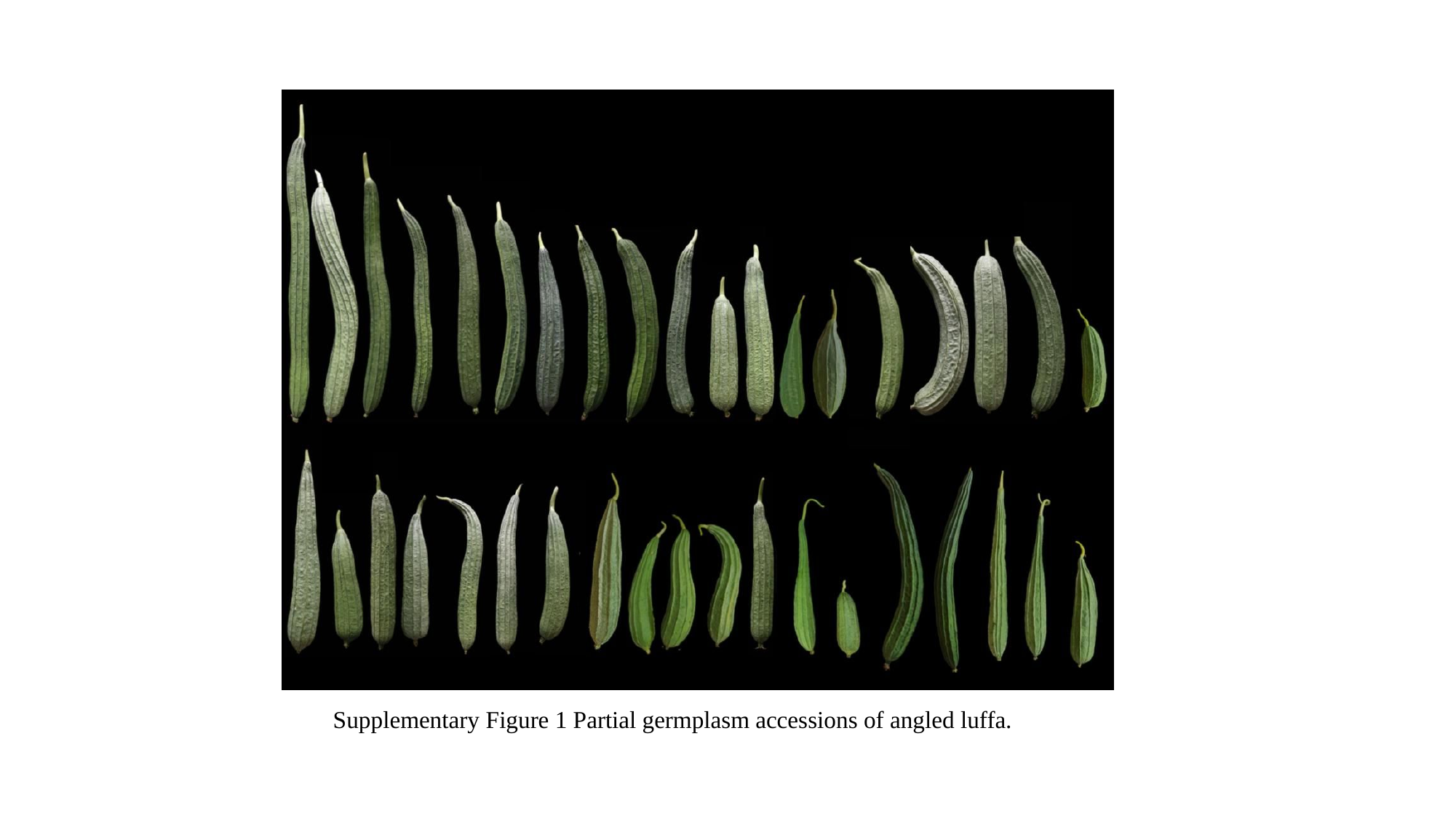

Supplementary Figure 1 Partial germplasm accessions of angled luffa.

## Slide 2
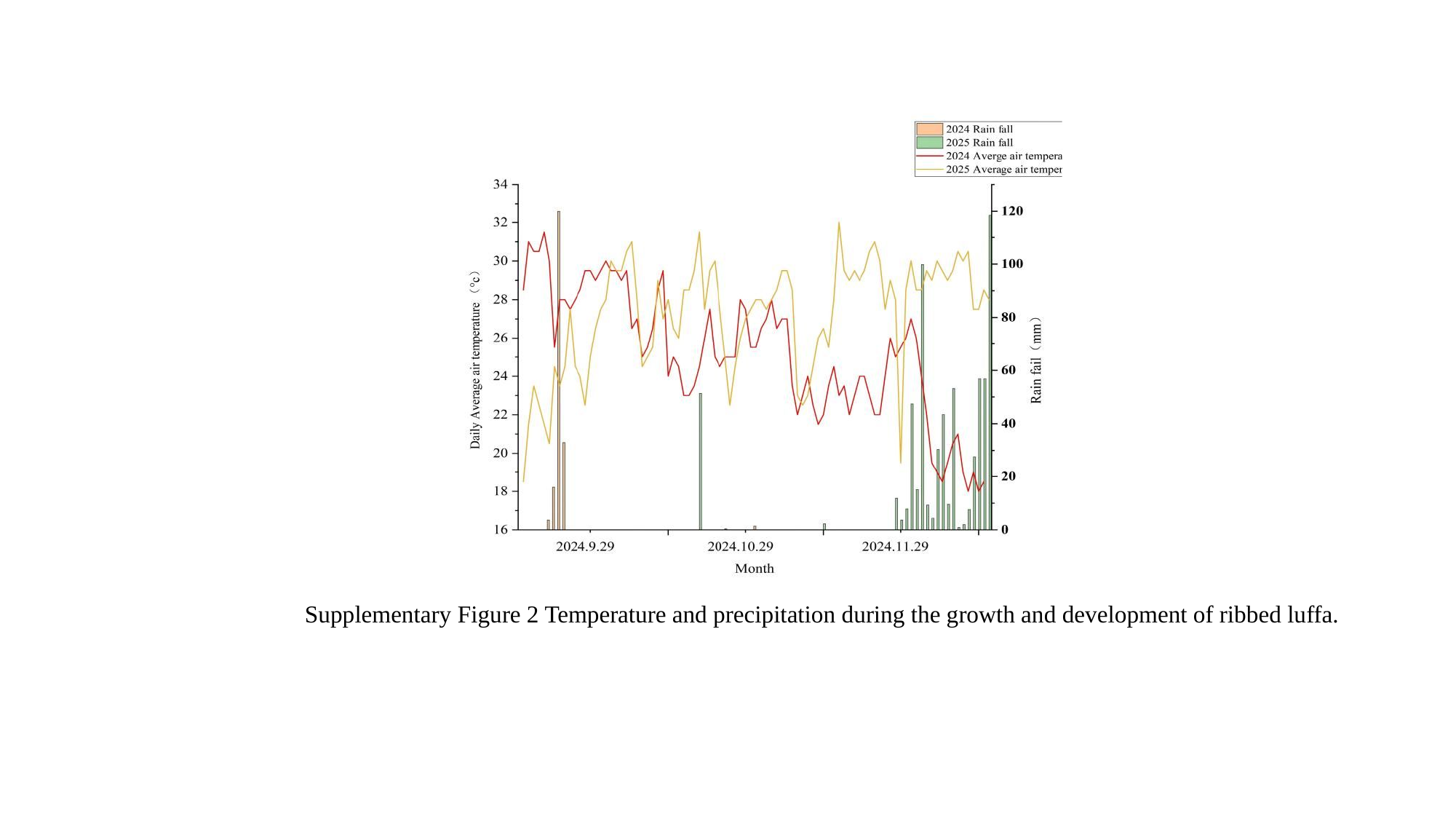

Supplementary Figure 2 Temperature and precipitation during the growth and development of ribbed luffa.

## Slide 3
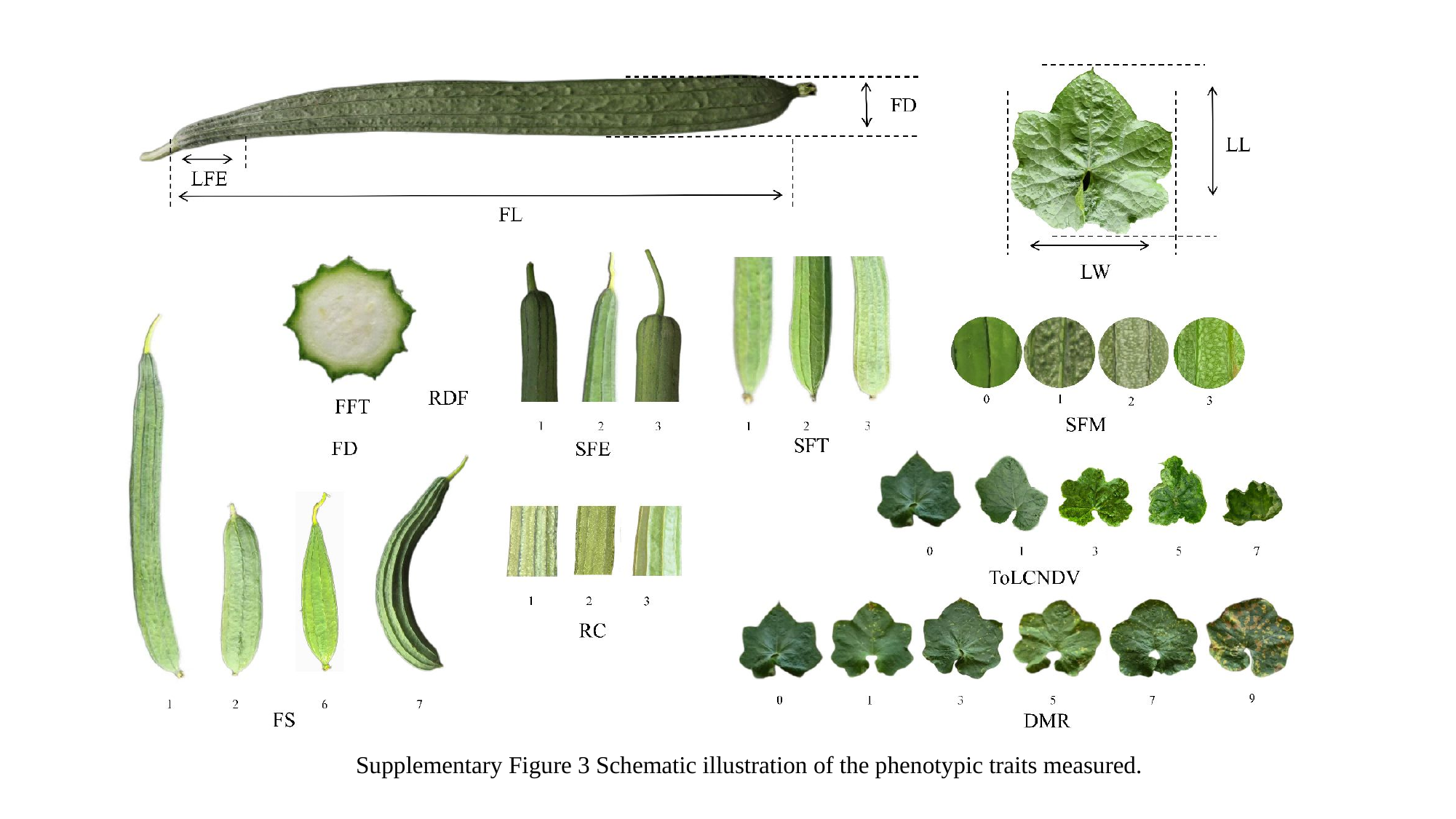

Supplementary Figure 3 Schematic illustration of the phenotypic traits measured.

## Slide 4
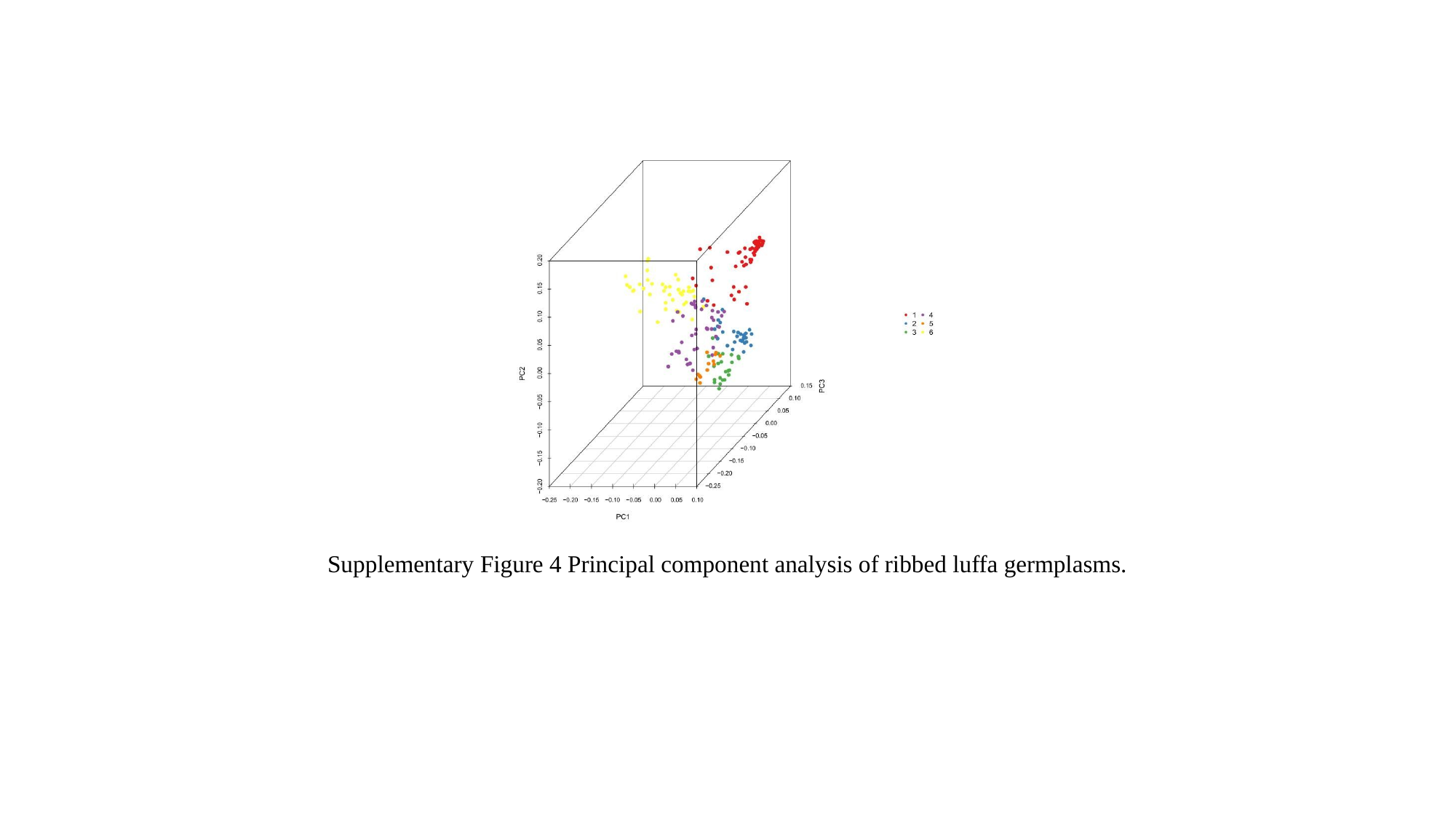

Supplementary Figure 4 Principal component analysis of ribbed luffa germplasms.

## Slide 5
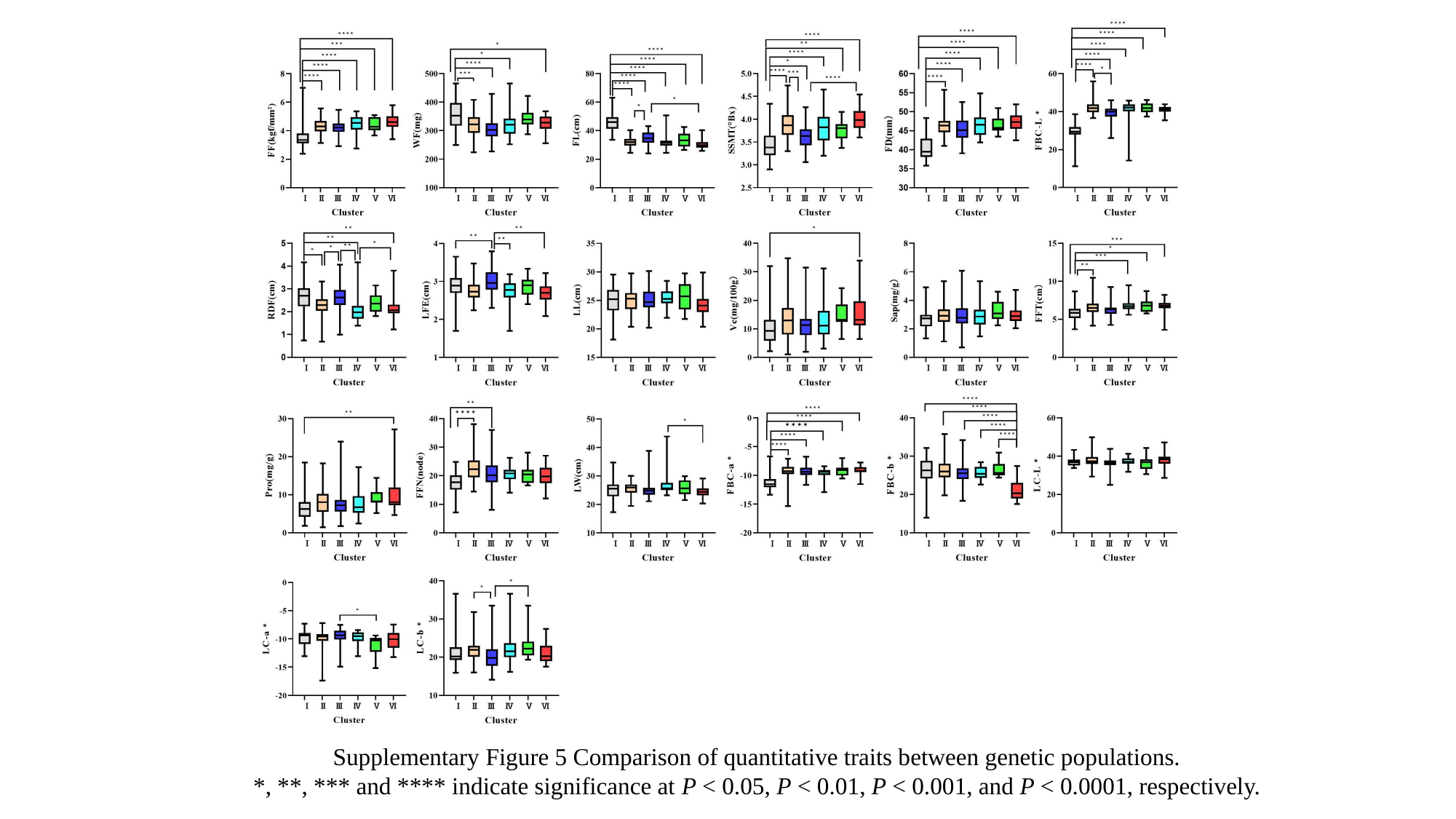

Supplementary Figure 5 Comparison of quantitative traits between genetic populations.
*, **, *** and **** indicate significance at P < 0.05, P < 0.01, P < 0.001, and P < 0.0001, respectively.
